# Establishing an electronic patient-reported outcome (ePRO) for patients with endometriosis and chronic pelvic pain: A pilot feasibility study

**DOI:** 10.1017/cts.2025.10155

**Published:** 2025-09-29

**Authors:** Elizabeth Randle, Raquel da Luz Dias, Allana Munro

**Affiliations:** 1 Department of Obstetrics & Gynaecology, IWK Health, Halifax, NS, Canada; 2 Department of Obstetrics & Gynaecology, Faculty of Medicine, Dalhousie University, Halifax, NS, Canada; 3 Department of Department of Women’s & Obstetric Anesthesia, IWK Health, Halifax, NS, Canada; 4 Department of Anesthesia, Pain Management and Perioperative Pain Medicine, Faculty of Medicine, Dalhousie University, Halifax, NS, Canada

**Keywords:** Endometriosis, chronic pelvic pain, patient-reported outcome measures, feasibility studies, patient experience

## Abstract

**Introduction:**

Endometriosis and chronic pelvic pain (CPP) are complex conditions that significantly impact quality of life. Few tools systematically capture patient-reported outcomes in this population. This pilot study evaluated patients’ experiences and the perceived usability of an electronic Patient-Reported Outcome (ePRO) tool to assess its feasibility in supporting a clinical data registry. Associations between demographic/clinical characteristics and ePRO usability were also explored.

**Methods:**

This prospective observational study included patients enrolled at a tertiary endometriosis and CPP clinic who completed a REDCap-based ePRO survey remotely. The survey included demographic items and 13 validated instruments assessing pain, psychological distress, sensory processing, and quality of life. Usability was evaluated through an Online Questionnaire-Experiences Survey (OQES), covering accessibility, completion experience, redundancy, and content relevance. Descriptive statistics, t-tests, and Hedges’ g were used for analysis; open-ended responses were thematically reviewed.

**Results:**

Fourteen patients were invited; 11 (78.6%) completed the full ePRO. Most found it easy to access (90.9%) with stable internet (100%). While 63.6% reported some redundancy, none reported discomfort, and 90.9% agreed the survey captured relevant experiences. Participants with higher Central Sensitization Inventory (CSI) and Generalized Anxiety Disorder-7 (GAD-7) scores were more likely to complete all items (*P* = 0.042 and .047). Those who did not perceive redundancy scored significantly higher on the Pain Catastrophizing Scale (*P* = .048) and Endometriosis Health Profile-30 (*P* = .016).

**Conclusion:**

The ePRO tool showed high feasibility. Patients with higher symptom burden were more likely to find it useful. Future improvements should reduce redundancy and clarify survey instructions.

## Introduction

Endometriosis and chronic pelvic pain (CPP) are complex, often debilitating conditions that affect millions of individuals worldwide. Endometriosis is defined by the presence of endometrial-like tissue outside the uterus, most commonly on pelvic structures such as the ovaries, bowel, and bladder, and less frequently on extra-pelvic sites including the lungs and pleura [1]. These lesions respond to hormonal fluctuations, leading to inflammation, fibrosis, and persistent pain [2]. Globally, more than 190 million people are affected, with prevalence estimates of up to 10% among those of reproductive age [3] and substantially higher rates among individuals presenting with pelvic pain, dysmenorrhea, or infertility [4].

Despite its prevalence and impact, endometriosis and CPP remain underdiagnosed and undertreated [5]. Its pathophysiology is poorly understood and likely multifactorial, involving hormonal, immunological, genetic [6], and psychosocial mechanisms [7], and overlapping with other gynecological causes such as adenomyosis, fibroids, pelvic inflammatory disease, and adhesions, as well as urological (e.g., bladder pain syndrome), gastrointestinal (e.g., irritable bowel syndrome), musculoskeletal (e.g., abdominal myofascial pain), and psychosocial factors (e.g., depression, anxiety, trauma history) [8–10]. In many cases, CPP involves centralized pain mechanisms, where the nervous system amplifies pain signals, exacerbating symptoms and complicating diagnosis and treatment [11]. Effective management requires more than symptom suppression – it calls for integrated, person-centered approaches that recognize the physical, emotional, and functional dimensions of the disease [12].

To address this complexity, multidisciplinary care models have emerged as the recommended standard [13,14]. These models bring together gynecology, mental health, pain management, physiotherapy, and other allied health disciplines to deliver comprehensive care. However, evaluating the effectiveness of such integrated approaches remains challenging due to a lack of high-quality, longitudinal patient data.

Collecting robust clinical and patient-reported data in this population presents several barriers. These include variability in symptom presentation, diagnostic delays, stigma, and the logistical difficulties of frequent in-person assessments [15]. Longitudinal registries offer a promising solution by enabling systematic data collection across timepoints. National initiatives such as the Endometriosis Pelvic Pain Interdisciplinary Cohort (EPPIC) in British Columbia have demonstrated the value of such registries in advancing clinical research and care [16,17].

IWK Health is in the early stages of building a local data registry through the Endometriosis and Chronic Pelvic Pain (E&CPP) Program [18]. As a first step, our team developed an electronic Patient-Reported Outcome (ePRO) tool to remotely capture standardized data on pain, psychological health, and quality of life. Electronic tools like ePROs offer numerous advantages, including scalability, convenience, and better integration with clinical workflows, particularly for patients with fluctuating symptoms or limited access to care [15,19,20].

To determine whether this ePRO tool could be successfully implemented in our local setting, we conducted a pilot feasibility study. Such studies are essential for identifying barriers, assessing usability, and informing adjustments before large-scale implementation [21]. This manuscript presents the development of the E&CPP ePRO tool and reports on findings from the pilot study evaluating its feasibility in supporting the launch of our local clinical registry.

The primary objective of this study is to describe the development of an ePRO tool and to assess its feasibility in supporting the establishment of the E&CPP clinical data registry. Secondary objectives include examining the associations between participants’ demographic and clinical characteristics and their reported experiences with the ePRO. Specifically, the study aims to address the following questions:

1. What is the adherence and engagement of patients with the ePRO questionnaire?
2. How do patients experience and perceive the usability of the ePRO tool, with a focus on accessibility, ease of completion, perceived redundancy, and content comprehensiveness?
3. What suggestions do patients offer to improve the ePRO system for future users and better align it with the needs of prospective patients and the creation of the E&CPP data registry?
4. Which demographic and clinical characteristics are associated with negative and positive perceived usability experiences with the ePRO?

## Methods

### Study design and settings

This prospective, observational pilot study evaluated the feasibility of an ePRO tool for patients enrolling at the IWK Health E&CPP Program. Based in Halifax, Nova Scotia, the E&CPP program specializes in the multidisciplinary management of endometriosis and CPP, providing specialized care for patients across the Atlantic Provinces of Canada.

The study was approved by the IWK Health Research Ethics Board (REB #1030268) as part of a wider initiative to support ongoing data collection for a local clinical data registry.

### ePRO development

The E&CPP ePRO tool was developed by an interdisciplinary team comprising gynecologists, an anesthesiologist, a nurse practitioner, a social worker, physiotherapists, and research scientists. This collaborative approach ensured that the tool was designed to provide a comprehensive assessment of endometriosis and CPP from multiple clinical perspectives.

The Research Electronic Data Capture (REDCap version 15.0.7) [22] system was adopted as the platform for the ePRO tool due to its secure, flexible, and customizable data management capabilities. REDCap features automated survey distribution, real-time data capture, encrypted data storage, and role-based access control, making it an ideal solution for implementing patient-reported outcomes in clinical settings [22]. The platform also enables longitudinal data tracking and remote access, supporting the long-term feasibility and scalability of the ePRO tool in establishing a local database for the E&CPP clinic, hosted by the IWK Health REDCap server.

The E&CPP ePRO tool was modeled after the EPPIC data registry [16,17], aligning with established data collection standards for individuals with endometriosis and CPP. This approach was intentionally designed to enhance interoperability and support future national collaboration by facilitating the aggregation and comparison of standardized datasets across clinical and research sites. Building on the EPPIC framework, the ePRO tool was further expanded by the E&CPP interdisciplinary team, integrating the distinct perspectives of each program discipline to ensure consistent symptom measurement and a comprehensive assessment of endometriosis and CPP from multiple clinical perspectives.

The onboarding (baseline) survey consisted of two sections. The first included eight demographic items designed to capture key background information, including age, province of residence, referral source, education level, employment status, and health insurance coverage. The second comprised 12 validated instruments commonly used to assess patient-reported symptoms, pain characteristics, psychological distress, and quality of life in this population, including the Short-Form McGill Pain Questionnaire (SF-MPQ) [23], Patient Health Questionnaire-9 (PHQ-9) [24], Generalized Anxiety Disorder-7 (GAD-7) [25], Pain Catastrophizing Scale (PCS) [26], Central Sensitization Inventory (CSI) [27], Pain Stages of Change Questionnaire (PSOCQ) [28], Tampa Scale for Kinesiophobia (TSK) [29], Rome Criteria and a Diagnostic Approach to Irritable Bowel Syndrome (ROME IV) [30], the Interstitial Cystitis Symptom Index (ICSI) and Problem Index (ICPI) [31], Female Sexual Distress Scale–Revised (FSDS-R) [32] and the Endometriosis Health Profile-30 (EHP-30) [33]. In total, the second part of the ePRO consisted of 210 items. All 12 instruments included in the ePRO have established psychometric properties in populations with chronic pelvic pain, endometriosis, and related conditions.

Upon completion of the validated instruments, REDCap generates a patient summary containing all scores and corresponding cut-off points. This summary is reviewed by the E&CPP clinic nurse and entered into the patient’s electronic medical record (EMR) in Accuro Cloud [34], the clinical platform used at IWK Health. This process ensures that patient-reported data are readily available to the care team before the initial assessment and to inform individualized treatment planning.

### Participants and data collection

Patients attending their initial appointment at the E&CPP Clinic were eligible to participate in the pilot study. Following their clinical consultation, a research assistant provided them with study information. Those interested in participating provided informed consent and were emailed a unique survey link granting access to the ePRO tool.

To evaluate the usability and patient experience with the ePRO tool, a structured Online Questionnaire-Experiences Survey (OQES) was included at the end of the ePRO baseline survey. The OQES comprised 15 items including multiple-choice, Likert-scale, and open-ended questions to capture patients’ perceptions regarding accessibility, ease of completion, perceived redundancy, and overall content relevance.

**Figure 1.**
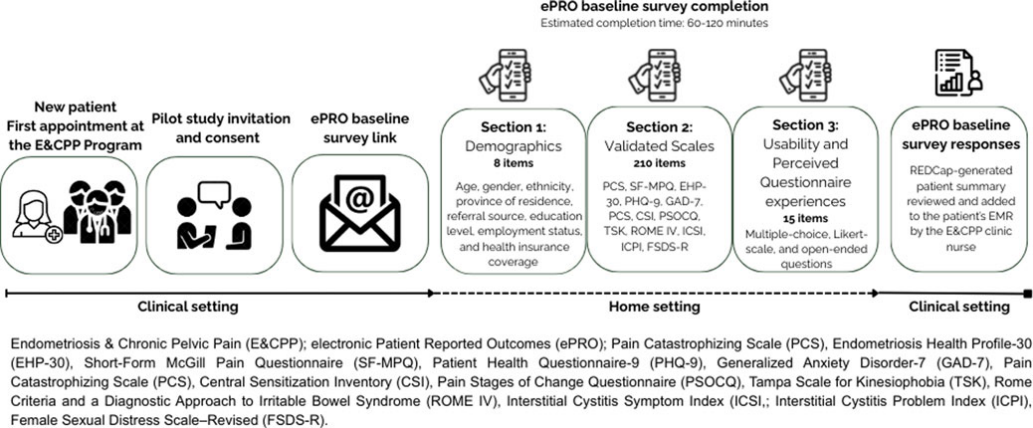
E&CPP ePRO workflow.

Participants were asked to complete the baseline survey within two weeks using their own devices. Participants did not receive any in-person or verbal guidance about how to complete the ePRO, other than the orientation provided in the email message with the survey link. Based on internal testing prior to implementation, the expected completion time for the full ePRO was approximately 60–120 minutes. A visual summary of the E&CPP ePRO workflow is provided in Figure 1.

### Outcome measures

Feasibility was measured within Teresi’s et al. guidelines for feasibility and pilot studies [21]. This study’s feasibility criteria outcomes were centered on the adherence and engagement of patients with the ePRO questionnaire, measured by the overall response and dropout rates of the onboard questionnaire sent to patients over the study period. The response rate was calculated as the proportion of participants who completed the full survey among those invited, while the dropout rate represented the proportion of participants who began but did not complete the survey.

Participants’ experience and perceived usability of the ePRO tool were measured across four key usability dimensions, including

- **Accessibility:** ease of opening the survey, resuming after a pause, accessing it online, and maintaining a consistent internet connection
- **Completion experience:** ability to answer all questions, completion pattern, time spent on the survey, perceived appropriateness of the time required, and level of distraction during completion
- **Perceived redundancy and comfort:** whether questions felt repetitive and whether participants experienced discomfort when reporting on certain topics
- **Content comprehensiveness:** perceived relevance and completeness of the questions in capturing symptoms, history, and experiences; appreciation for being asked about these topics; and belief that the information would support informed healthcare decisions.

### Data analysis

Data were analyzed using SPSS version 28.0 [35]. Descriptive statistics, including counts, frequencies, percentages, means, and standard deviations, were used to summarize participants’ demographic and clinical characteristics. Independent samples *t*-tests were conducted to compare demographic and clinical variables across groups defined by usability responses. Group comparisons were conducted for mean age and mean scores on validated clinical scales. To accommodate the small sample size, OQES responses were dichotomized. Likert-scale items were recoded into “agreement” (combining “Agree” and “Strongly Agree”) versus “non-agreement” (“Strongly Disagree,” “Disagree,” and “Neutral”), and other multi-category variables were binarized based on conceptually meaningful groupings. Variables with no response variability were excluded from statistical analyses.

Statistical significance was determined using two-tailed *p*-values (*P* ≤ 0.05). Effect sizes were reported using Hedges’ g, interpreted as small (≥ 0.2), moderate (≥ 0.5), or large (≥ 0.8) [36]. Effect sizes and 95% confidence intervals were used to assess the magnitude and potential clinical relevance of group differences. All analyses were based on complete case data; participants with missing values for any comparison variable were excluded from that analysis.

Qualitative data from open-ended responses were analyzed using conventional content analysis. Responses were independently reviewed and coded to identify common themes, including perceived challenges, user experience, and suggestions for improvement.

### Sample size considerations

Consistent with published guidance for pilot feasibility studies [21], the sample size was determined pragmatically, based on available resources, participant flow, and the number of patients needed to reasonably assess feasibility outcomes. Small sample sizes are acceptable and methodologically appropriate for this type of study, where the goal is to evaluate processes rather than achieve statistical power. This study was intentionally designed as a pilot feasibility evaluation of the ePRO tool within the operational context of the E&CPP clinic – which operates one day per week and was in the early stages of establishing its services during the study period – and not for the general population with chronic pelvic pain. The primary objective was to assess usability, acceptability, and integration of the ePRO into the clinic’s workflow prior to broader rollout rather than to make population-level inferences.

## Results

### Participant characteristics and ePRO completion rates

Fourteen (14) patients were invited to participate in the pilot feasibility study. The demographic section of the ePRO achieved a 100% response rate. On average, participants were Caucasian females, 35 (±8) years of age, residing in Nova Scotia, with a post-secondary level of education. Most were employed, referred to the E&CPP program by a family physician, and covered by private drug insurance. A detailed summary of participant demographics is provided in Table 1.

**Table 1.** Sociodemographic and clinical characteristics

Patient Characteristics		Frequency *n*	Percentage %	
**Gender-Sex Concordance**	Yes	13	100%	
	No	No	0	0%
**Province of Origin**	Nova Scotia	10	71.42%	
	New Brunswick	New Brunswick	2	14.28%
	Prince Edward Island	Prince Edward Island	2	14.28%
**Referral Source**	Family doctor	5	38.46%	
	Gynecologist	4	30.76%	
	Nurse practitioner	Nurse practitioner	2	15.38%
	Other	Other	2	15.38%
**Ethnicity**	White/Caucasian	14	100%	
	Other^*^	Other^*^	0	–
**Education Level**	Elementary/junior high school	0	–	
	High school	High school	5	35.71%
	Post-secondary school	Post-secondary school	9	64.28%
**Employment Status**	Student	1	7.69%	
	Employed (Full/part-time)	Employed (Full/part-time)	9	69.23%
	Unemployed	Unemployed	0	–
	Disability/Sick leave	Disability/Sick leave	3	23.07%
**Drug Insurance Coverage**	None	1	7.14%	
	Public (Pharmacare, Prescription Drug Program)	3	21.42%	
	Private	10	71.42%	

* Other ethnicity options included Latino-American, Black (African, Afro-American, Afro-Caribbean), Aboriginal (First Nations, Inuk or Metis), South Asian (India, Pakistan, Sri Lanka), Filipino, Arab, Chinese, Southeast Asian (Vietnam, Cambodia, Malaysia, Laos), Western Asian (Iran, Afghanistan), Korean, Japanese, and Other.

Of the 14 participants, 11 (78.6%) completed the second component, which included the validated scales. Among the three participants who did not complete the second component, two completed only the first two instruments (PCS and SF-MPQ) before stopping, and one left the SF-MPQ incomplete. Additionally, one participant did not complete all items of the FSDS-R, which prevented calculation of final scores for this scale. Given the small number of non-completers (*n* = 3), statistical comparisons of socio-demographic characteristics between completers and non-completers were not conducted, as meaningful analysis was not feasible.

Clinical characteristics of the participants, assessed through validated instruments embedded in the ePRO, are detailed in Table 2. Findings indicated elevated levels of pain-related cognitive and sensory processing impairments among participants. Scores across the PCS, SF-MPQ, TSK, and CSI reflected high levels of pain catastrophizing, moderate to severe pain intensity, kinesiophobia, and central sensitization. Health-related quality of life, as measured by the EHP-30, was moderately impaired. PSOCQ scores suggested limited readiness for active self-management, with most participants classified in the precontemplation or contemplation stages, few in the maintenance and none in the action stage.

**Table 2.** Clinical characteristics of participants

Domain	Scale	Threshold Score	Mean Score (SD)	Min/Max Score	Total (*N*)	Frequency within/above cut-off score categories % (*n*)
**Pain-related cognitive and sensory processing**	**PCS**	≥30	30.62 (±13.85)	3/50	14	50% (7)
	**SF-MPQ**	**Overall** ≤17 mild 18–27 moderate ≥28 severe	27.08 (±7.34)	17/38	12	Mild: 8.33% (1) Moderate: 58.33% (7) Severe: 33.33% (4)
		**Sensory** ≤14 mild 15–22 moderate ≥23 severe	18.67 (±4.27)	13/26		Mild: 16.66% (2) Moderate: 58.33% (7) Severe: 25% (3)
		**Affective** ≤4 mild 5–8 moderate ≥9 severe	7.58 (±3.03)	3/12		Mild: 16.66% (2) Moderate: 50% (6) Severe: 33.33% (4)
	**TSK**	>37	43.27 (±6.83)	32/55	11	81.81% (9)
	**CSI**	≥40	60.64 (±20.32)	31/94	11	81.81% (9)
	**EHP-30**	0 to 100*	59.65 (±20.71)	23.4/100	11	–
	**PSOCQ**	Precontemplation	3.44 (±0.80)	2.14/4.86	11	27.27% (3)
		Contemplation	3.82 (±0.34)	3.30/4.40		27.27% (3)
		Action	3.80 (±0.25)	3.33/4.17		0
		Maintenance	3.70 (±0.62)	2.29/4.14		45.45% (5)
**Psychological distress**	**PHQ-9**	≥10	15.55 (±8.66)	2/27	11	72.72% (8)
	**GAD-7**	≥10	10.73 (±7.10)	0/21	11	54.54% (6)
**Somatic symptoms**	**ICSI**	0 to 20*	7.45 (±4.16)	2/14	11	–
	**ICPI**	0 to 16*	5.55 (±4.48)	0/13	11	–
	**Rome IV**	≥ 2	–	–	11	36.36% (4)
**Sexual functioning**	**FSDS-R**	≥11	27.36 (±14.56)	2/47	10	80% (8)

Pain Catastrophizing Scale (PCS), Short-Form McGill Pain Questionnaire (SF-MPQ), Tampa Scale of Kinesiophobia (TSK), Central Sensitization Inventory (CSI), Endometriosis Health Profile-30 (EHP-30), Pain Stages of Change Questionnaire (PSOCQ), Patient Health Questionnaire-9 (PHQ-9), Generalized Anxiety Disorder-7 (GAD-7), Interstitial Cystitis Symptom Index (ICSI), Interstitial Cystitis Problem Index (ICPI), Female Sexual Distress Scale–Revised (FSDS-R). *No cut-off established.

Psychological distress was prominent, with the majority of participants meeting clinical thresholds for depression and anxiety on the PHQ-9 and GAD-7, respectively. Somatic symptom burden, measured by the ICSI and ICPI, was mild to moderate. A notable proportion of participants screened positive for IBS symptoms using the Rome IV criteria, and sexual distress was common, with most respondents exceeding the clinical threshold on the FSDS-R.

### Perceived usability and experience with the ePRO

The completion rate of the third and last part of the ePRO, aimed at evaluating the perceived usability and experience of respondents through the OQES was 78.6% (*n* = 11). Overall, participants reported a high level of usability and satisfaction with the ePRO system (Table 3). Most found the survey easy to access and resume, with minimal technical issues or interruptions during completion. The majority completed the survey in one sitting, and all participants rated the time required as appropriate. No distractions or discomfort were reported. While some participants perceived repetition in the survey content, most agreed that it covered all relevant symptoms and experiences. Respondents expressed appreciation for being asked about their health history and endorsed the value of their responses in informing future clinical care.

**Table 3.** Perceived Usability and experience with the ePRO

Theme	Question	Response Options	Response Distribution % (*n*)
**Accessibility**	Opening the survey was easy (*n* = 11)	Strongly disagree	0% (0)
		Disagree	9.1% (1)
		Neither agree nor disagree	0% (0)
		Agree	27.3% (3)
		Strongly agree	63.6% (7)
	It was easy to pick up where you left. (*n* = 11)	Yes	72.72% (8)
		No	27.27% (3)
	It was easy to get online to complete the surveys. (N = 11)	Yes	90.9% (1)
		No	9.1% (1)
	I had consistent internet connection when completing the surveys. (*n* = 11)	Yes	100% yes
		No	0% (0)
**Completion Experience**	Were you able to answer all the questions? (*n* = 11)	Yes	72.7% (8)
		No	27.3% (3)
	When completing the survey, I: (*n* = 11)	Completed all items in one sitting	63.6% (7)
		Left the survey and came back to it one or more times	36.4% (4)
	How many times did you leave and return to the survey? (*n* = 11)	0	54.5% (6)
		1	36.4% (4)
		≥2	9.1% (1)
	How much time did you spend completing the surveys? (*n* = 10)	<60 min	60% (6)
		1–2 hours	30% (3)
		>2 hours	10% (1)
	I found the time needed to complete the scales to be (*n* = 11)	Too much	0% (0)
		A lot	0% (0)
		Good	100% (11)
		Short	0% (0)
	I was distracted by events or other people while completing the surveys (*n* = 10)	Yes	0% (0)
		No	100% (10)
**Redundancy and Comfort**	I was uncomfortable reporting on some of the things asked in the survey (*n* = 11).	Yes	0% (0)
		No	100% (11)
	I felt I was asked the same thing or things multiple times (*n* = 11).	Yes	63.6% (7)
		No	36.4% (4)
**Content and Comprehensive-ness**	I am glad the E&CPP program at the IWK is asking me about my history and symptoms (*n* = 11).	Strongly disagree	0% (0)
		Disagree	0% (0)
		Neither agree nor disagree	9.1% (1)
		Agree	27.3% (3)
		Strongly agree	63.6% (7)
	I hope the information I give will be used to help make good healthcare decisions with the team at the IWK (*n* = 11).	Strongly disagree	0% (0)
		Disagree	0% (0)
		Neither agree nor disagree	0% (0)
		Agree	27.3% (3)
		Strongly agree	72.7% (8)
	The surveys covered all symptoms, history and experiences relevant to why I am seeking healthcare from the IWK E&CPP program (*n* = 11).	Yes	90.9% (10)
		No	9.1% (1)

Endometriosis & Chronic Pelvic Pain (E&CPP).

### Demographic and clinical correlates of ePRO usability and user experience

Four (4) usability and user experience variables presented sufficient variability in responses and adequate group sizes allowing correlational analysis: three (3) from the completion experience domain (completion of all questions, number of sittings, and time to completion) and one (1) from the redundancy and comfort domain (perceived redundancy).

In the completion experience domain, participants who completed all survey items had significantly higher mean scores on the CSI and GAD-7 compared to those who did not. In the redundancy and comfort domain, participants who did not perceive redundancy in the survey scored significantly higher on the PCS and EHP-30 (Table 4). No statistically significant differences were found across other clinical variables (Supplementary Material, Appendix 1).

**Table 4.** Independent *t*-test for significant demographic and clinical variables by usability domains

Usability domain: Completion Experience (Completed all questions)								
**Variable**	**Group**	**Mean ± SD**	**Mean Diff**	**95% CI (Mean Diff)**	**T (df)**	***P***	**g**	**95% CI (g)**
CSI	Yes (*n* = 8)	68.0 ± 18.6	−27.00	−52.71, -1.28	−2.37 (9)	0.042	−1.46	−2.82, -0.10
	No (*n* = 3)	41.0 ± 7.6						
GAD-7	Yes (*n* = 8)	13.25 ± 6.5	−9.25	−18.34, -0.15	−2.30	0.047	−1.42	−2.76, -0.01
	No (*n* = 3)	4.0 ± 3.6						
**Usability domain: Redundancy and comfort (Redundancy)**								
PCS	Yes (*n* = 7)	25.0 ± 14.34	17.75	0.20, 35.29	2.28 (9)	0.048	1.31	0.11, 2.55
	No (*n* = 4)	42.75 ± 6.94						
EHP-30	Yes (*n* = 7)	49.20 ± 15.84	28.75	6.66, 50.83	2.94 (9)	0.016	1.68	0.29, 3.01
	No (*n* = 4)	77.0 ± 15.02						

Central Sensitization Inventory (CSI), Generalized Anxiety Disorder-7 (GAD-7), Endometriosis Health Profile-30 (EHP-30), Pain Stages of Change Questionnaire (PSOCQ).

Two usability variables within the completion experience domain (number of sittings and time to completion) presented borderline findings (*P* ≥0.05 and ≤ 0.10) and large effect sizes (Hedges’ *g* ≥ 0.80) (Supplementary Material, Table 2 and Table 3). For number of sittings, the largest mean difference was in age, with participants completing the survey in multiple sittings being older (41.25 ± 6.60) than those completing it in a single sitting (31.00 ± 8.92). Although not statistically significant (*P* = .078), the associated effect size was large (Hedges’ g = –1.39, 95% CI [–2.35, 0.12]), indicating a potentially meaningful difference despite wide confidence intervals. Similarly, for time to completion, participants who required more than one hour to complete the survey were older (41.25 ± 6.60) than those who completed it within one hour (30.17 ± 9.47). This difference approached statistical significance (*P* = .078) and was associated with a large effect size (Hedges’ g = –1.17, 95% CI [–2.42, 0.12]), suggesting a possible relationship between age and survey duration. No statistically significant differences were found across clinical variables (Supplementary Material, Appendix 1).

### Qualitative data on the ePRO usability and user experience

The frequency of participant responses to survey questions, along with the emergent themes and representative comments is summarized in Table 5. Patients reported difficulties with recalling specific medical history details such as dates of surgeries and age at symptom onset. One patient experienced a temporary technical error while completing the survey. While some patients perceived questions are being repetitious, particularly regarding topics related to pain and mental health, no participants reported discomfort with any survey content. Other comments focused on the need for additional details about medication use, more frequent follow-up surveys, need for continued engagement with the research, and the need for clearer instructions prior to survey initiation.

**Table 5.** Frequency of responses by survey question, emergent themes and participant comment sample

Survey Question	Total Responses	Emergent Themes	Participant comment
Challenges with the survey	3	Complexity of medical history input and recall difficulty	*“Trying to remember exact ages and years of when events happened, especially worst pain age.”*
Challenges leaving and returning to the survey	0	No challenges leaving and returning to the survey reported.	–
Online logistical challenges	1	Technical glitches (e.g., error messages)	*“I kept getting an error msg but it fixed itself.”*
Perceptions of redundancy	7	Question repetition	*“I did feel I was asked the same question a few times but I do understand it was to ensure accuracy of the answer.”*
Uncomfortable content	0	No uncomfortable content reported	–
Views on comprehensiveness	1	Suggestion for depth and inclusion of relevant topics	“*In regards to hormonal medication history it may be good to ask about side effects experienced and if side effects were the reason for discontinued use. Or if medication because less effective over time. I’ve had bad experiences with most medication and more recently the medication I’ve been on long term has stopped working.”*
Suggestions for follow-up	1	Intertest in participating in the data registry and research	“*I would be happy to participate more regularly, as in every 3-6 months, depending on length of the survey(s). I am deeply appreciative of the research being done in this area.”*
General feedback	2	Need for clear instructions prior survey completion	*“…I would suggest sending an email prior to letting the patient know what information will be required for example medication dates of surgeries etc. As well as an option for that patient to request that information if they do not have it themselves otherwise they won’t be able to complete the survey or the information will be accurate.”*

## Discussion

This pilot feasibility study evaluated the development and initial implementation of a REDCap-based ePRO tool designed to support the establishment of a clinical data registry for endometriosis and CPP patients. Overall, the findings demonstrate that the ePRO tool is a feasible and acceptable method for collecting patient-reported outcomes in this population. The high rate of consent, survey initiation, and completion, as well as the positive feedback regarding accessibility, content relevance, and user satisfaction, support its potential integration into routine clinical care and research workflows.

### Completion rate and accessibility

Previous studies have documented considerable variability in patient adherence to ePRO completion, with reported rates ranging from approximately 40% to 60% in clinical settings [37,38], contrasting with the nearly 80% completion rate observed in the present study. Evidence on the patient-level factors influencing ePRO response rates remains limited. However, lower socioeconomic status and rural residence have been identified as predictors of reduced internet access and lower e-health literacy, both of which may negatively affect ePRO engagement [39]. This pattern was not reflected in our sample, where all participants reported stable internet access and the majority indicated that the tool was easy to access, navigate, and complete online. Qualitative feedback supported this finding, with no participants reporting difficulty returning to the survey after a break, and only one participant noting a brief technical error. These findings suggest good overall accessibility of remote, self-administered ePRO completion using REDCap.

In this pilot, participants reported high education levels, as most had post-secondary education. While education level was not significantly associated with ePRO completion in our sample, higher education has been linked in prior research to greater confidence using online tools, which could contribute to engagement [40,41].

Although initial completion rates and overall accessibility were high, it is important to acknowledge that the ePRO tool evaluated in this study was designed to support a longitudinal data registry for patients in the E&CPP program. Sustained adherence to follow-up surveys is essential to ensure that the registry remains current, comprehensive, and clinically relevant. In longitudinal applications, ePRO completion rates often decline over time, with some studies reporting rates as low as 25% one year after baseline intake [42,43]. In contrast, other evidence indicates that completion rates may increase over time as both patients and clinicians become more familiar with the tool and its integration into routine care workflows [42].

### Completion experience

Regarding completion experience, one notable finding was that older participants in our study were more likely to require multiple sittings and longer durations to complete the ePRO, suggesting increased time burden rather than greater engagement. This contrasts with previous studies that have associated older age – particularly among the baby boomer generation – with higher ePRO response rates and more consistent participation [43,44]. This necessitates consideration since an additional hour of survey time increases the probability that a respondent skips a question by 10%–64% [45]. Despite this age-related difference in completion pattern, the overall participant experience was positive, with the majority reporting that the survey was easy to complete and that the time required was appropriate and consistent with the established expected completion time for the full ePRO indicating good overall completion experience of the tool across the sample.

However, challenges in the completion experience appeared in the qualitative responses, where participants noted challenges with recalling specific medical history details such as the dates of surgeries or age at symptom onset. This is an important consideration given that the potential for recall bias, when participants erroneously provide responses that depend on their ability to recall past events, leading to an error [46]. Clearer instructions at the beginning of the survey were also suggested to improve the overall experience [47]. These comments point to the importance of user preparation and pre-survey guidance, particularly when memory-based recall is required.

Completion experience was positively associated with higher CSI scores, as participants with greater symptom burden were more likely to complete all survey items. This suggests that individuals experiencing more severe symptoms may be more motivated to engage fully with comprehensive assessments. These findings are consistent with prior research indicating that patients with advanced disease stages or more intense symptoms tend to demonstrate higher completion rates and lower attrition [48]. Similarly, participants with elevated GAD-7 scores were significantly more likely to complete the questionnaire, aligning with previous studies showing greater retention among individuals with anxiety disorders compared to those without [49].

### Redundancy and comfort

Redundancy was frequently reported by participants in this study and may be viewed as both a positive and negative aspect of the ePRO tool. Commonly associated with “question fatigue,” redundancy arises when similar questions are asked in different ways and is often linked to poorly designed surveys [50]. On one hand, it can lead to increased respondent burden, reduced data quality, and lower completion rates [50,51]. On the other hand, redundancy may enhance measurement reliability through the “redundancy gain” effect, in which overlapping items improve cognitive processing, response accuracy, and consistency [52]. In this case, perceived redundancy likely stemmed from the inclusion of multiple validated instruments assessing related constructs. While improvements in survey logic and item presentation could potentially reduce perceived repetition, altering validated measures is not feasible without compromising their psychometric integrity and clinical utility.

Qualitative responses supported this interpretation, since the majority of participants described perceived redundancy, particularly in questions related to pain and mental health. However, no participants reported discomfort with any content, reinforcing that the repetition, although noted, did not diminish acceptability. Interestingly, individuals who did not report redundancy scored significantly higher on the PCS and EHP-30, suggesting that those experiencing greater distress may find repetitive items more relevant or necessary, resulting in a lower likelihood of perceiving it as repetitive.

### Content and comprehensiveness

Most participants agreed that the ePRO tool captured all relevant symptoms and experiences, and all expressed appreciation for being asked about their history and symptoms. This supports the notion that repetition may have promoted greater engagement and data completeness, consistent with the redundancy gain effect. The absence of reported discomfort also suggests that the content was appropriate and sensitively worded. Qualitative feedback highlighted the value of including more detailed questions about hormonal medication indicating areas for future refinement. The interest expressed in continued participation further underscores the tool’s acceptability and perceived clinical relevance.

### Strengths and limitations

This study’s strengths include the development of an ePRO tool through interdisciplinary collaboration, integrating expertise from the E&CPP team to ensure content relevance across the biopsychosocial dimensions of chronic pelvic pain and alignment with clinical and patient-centered care priorities. The mixed methods approach allowed for a deeper understanding of usability and generated actionable recommendations for refinement, despite the small sample size, which limits generalizability and reduces statistical power for subgroup analyses.

The sample’s homogeneity further limits applicability to more diverse populations and may overlook usability challenges experienced by individuals from different backgrounds. Additionally, the study focused solely on the baseline onboarding survey, without evaluating longitudinal feasibility. As a result, conclusions regarding sustained engagement and long-term registry implementation cannot be drawn. Finally, the survey was completed independently and without real-time technical support, which – while aligned with the intended use – may have influenced usability, particularly for participants with limited digital literacy.

## Conclusions

This pilot study demonstrated that the REDCap-based ePRO tool is a feasible and acceptable method for capturing patient-reported outcomes in individuals with endometriosis and CPP, with high completion rates and positive user feedback supporting its integration into clinical care and registry development. Participants with greater central sensitization and anxiety were more likely to engage fully with the tool and perceive it as relevant, whereas those with lower quality of life and higher pain catastrophizing were more likely to perceive redundancy. Further research should focus on assessing longitudinal use and improving accessibility through clearer instructions, a pre-survey checklist, and optimized survey logic to reduce perceived redundancy, and user support to ensure broad applicability and sustained engagement. Ultimately, translating this knowledge to other clinical contexts may help clinics in different regions feel more confident in establishing similar databases and foster opportunities for national collaboration in standardized data collection for endometriosis and chronic pelvic pain.

## Supporting information

10.1017/cts.2025.10155.sm001 Randle et al. supplementary material Randle et al. supplementary material

## Supplementary material

The supplementary material for this article can be found at https://doi.org/10.1017/cts.2025.10155.

## References

[1] Takebayashi A , Kimura F , Kishi Y , et al. The association between endometriosis and chronic endometritis. PLoS One. 2014;9:e88354. doi: 10.1371/journal.pone.0088354. 24558386 PMC3928198

[2] Wang Y , Nicholes K , Shih IM. The origin and pathogenesis of endometriosis. Annu Rev Pathol. 2020;15:71–95. doi: 10.1146/annurev-pathmechdis-012419-032654. 31479615 PMC7980953

[3] World Health Organization. Endometriosis. (https://www.who.int/news-room/fact-sheets/detail/endometriosis) Accessed April 2, 2025.

[4] Falcone T , Flyckt R. Clinical management of endometriosis. Obstet Gynaecol. 2018;131:557–571. doi: 10.1097/AOG.0000000000002469. 29420391

[5] Mehedintu C , Plotogea MN , Ionescu S , Antonovici M. Endometriosis still a challenge. J Med Life. 2014;7:349–357. 25408753 PMC4233437

[6] Marquardt RM , Tran DN , Lessey BA , Rahman MS , Jeong JW. Epigenetic dysregulation in endometriosis: Implications for pathophysiology and therapeutics. Endocr Rev. 2023;44:1074–1095. doi: 10.1210/endrev/bnad020. 37409951 PMC10638603

[7] Kalfas M , Chisari C , Windgassen S. Psychosocial factors associated with pain and health-related quality of life in endometriosis: A systematic review. Eur J Pain. 2022;26:1827–1848. doi: 10.1002/ejp.2006. 35802060 PMC9543695

[8] Berkley KJ , Riel R. Chronic pelvic pain in females beyond the basics. UpToDate. (https://www.uptodate.com/contents/chronic-pelvic-pain-in-females-beyond-the-basics/print) Accessed February 22, 2015.

[9] Szypłowska M , Tarkowski R , Kułak K. The impact of endometriosis on depressive and anxiety symptoms and quality of life: A systematic review. Front Public Health. 2023;11:1230303. doi: 10.3389/fpubh.2023.1230303. 37744486 PMC10512020

[10] Hemmert R , Schliep KC , Willis S , et al. Modifiable lifestyle factors and risk for incident endometriosis. Paediatr Perinat Epidemiol. 2019;33:19–25. doi: 10.1111/ppe.12516. 30307628 PMC6353682

[11] Kaya S , Hermans L , Willems T , Roussel N , Meeus M. Central sensitization in urogynaecological chronic pelvic pain: A systematic literature review. Pain Physician. 2013;16:291–308. 23877446

[12] Jarrell JF , Vilos GA , Allaire C , et al. Consensus guidelines for the management of chronic pelvic pain. J Obstet Gynaecol Can. 2005;27:781–826. doi: 10.1016/S1701-2163(16)30732-0. 4 De Graaff AA, D’Hooghe TM, Dunselman GA, Dirksen CD, Hummelshoj L, Consortium WE, Simoens S. 16287011

[13] Agarwal SK , Foster WG , Groessl EJ. Rethinking endometriosis care: Applying the chronic care model via a multidisciplinary program for the care of women with endometriosis. Int J Womens Health. 2019;11:405–410. doi: 10.2147/IJWH.S207373. 31413643 PMC6661982

[14] Pickett C , Foster WG , Agarwal S. Current endometriosis care and opportunities for improvement. Reprod Fertil. 2023;4:e220091. doi: 10.1530/RAF-22-0091. 37402150 PMC10448566

[15] Nicolas-Boluda A , Oppenheimer A , Bouaziz J , Fauconnier A. Patient-reported outcome measures in endometriosis. J Clin Med. 2021;10:5106. doi: 10.3390/jcm10215106. 34768627 PMC8585017

[16] Xu Y , Deng Z , Fei F , Zhou S. An overview and comprehensive analysis of interdisciplinary clinical research in endometriosis based on trial registry. IScience. 2024;27:109298. doi: 10.1016/j.isci.2024.109298. 38455973 PMC10918267

[17] Endometriosis Pelvic Pain Interdisciplinary Cohort (EPPIC) Registry. (https://yonglab.med.ubc.ca/eppic/endometriosis-pelvic-pain-interdisciplinary-cohort-eppic-data-registry/) Accessed March 20, 2025.

[18] IWK Health, Endometriosis & Chronic Pelvic Pain Program. (https://www.iwkecpp.ca/) Accessed March 26, 2025.

[19] Gwaltney CJ , Shields AL , Shiffman S. Equivalence of electronic and paper-and-pencil administration of patient-reported outcome measures: A meta-analytic review. Value Health. 2008;11:322–333. 18380645 10.1111/j.1524-4733.2007.00231.x

[20] Boutib A , Azizi A , Youlyouz-Marfak I , et al. Electronic patient-reported outcome measures (ePROs) as tools for assessing health-related quality of life (HRQoL) in women with gynecologic and breast cancers: a systematic review. Digit Health. 2024;10. doi: 10.1177/20552076241297041. PMC11552042 39529915

[21] Teresi JA , Yu X , Stewart AL , Hays RD. Guidelines for designing and evaluating feasibility pilot studies. Med Care. 2022;60:95–103. doi: 10.1097/MLR.0000000000001664. 34812790 PMC8849521

[22] Harris PA , Taylor R , Thielke R , Payne J , Gonzalez N , Conde JG. Research electronic data capture (REDCap)—a metadata-driven methodology and workflow process for providing translational research informatics support. J Biomed Inform. 2009;42:377–381. doi: 10.1016/j.jbi.2008.08.010. 18929686 PMC2700030

[23] Melzack R. The short-form McGill Pain Questionnaire. Pain. 1987;30:191–197. doi: 10.1016/0304-3959(87)91074-8. 3670870

[24] Kroenke K , Spitzer RL , Williams JB. The PHQ-9: validity of a brief depression severity measure. J Gen Intern Med. 2001;16:606–613. 11556941 10.1046/j.1525-1497.2001.016009606.x PMC1495268

[25] Spitzer RL , Kroenke K , Williams JB , Löwe B. A brief measure for assessing generalized anxiety disorder: The GAD-7. Arch Intern Med. 2006;166:1092–1097. doi: 10.1001/archinte.166.10.1092. 16717171

[26] Sullivan MJL , Bishop SR , Pivik J. The pain catastrophizing scale: development and validation. Psychol Assess. 1995;7:524–532. doi: 10.1037/1040-3590.7.4.524.

[27] Mayer TG , Neblett R , Cohen H , et al. The development and psychometric validation of the Central Sensitization Inventory (CSI). Pain Pract. 2012;12:276–285. 21951710 10.1111/j.1533-2500.2011.00493.x PMC3248986

[28] Kerns RD , Rosenberg R , Jamison RN , Caudill MA , Haythornthwaite J. Readiness to adopt a self-management approach to chronic pain: The Pain Stages of Change Questionnaire (PSOCQ). Pain. 1997;72:227–234. doi: 10.1016/s0304-3959(97)00038-9. 9272807

[29] Roelofs J , Goubert L , Peters ML , Vlaeyen JW , Crombez G. The Tampa scale for Kinesiophobia: Further examination of psychometric properties in patients with chronic low back pain and fibromyalgia. Eur J Pain. 2004;8:495–502. doi: 10.1016/j.ejpain.2003.11.016. 15324781

[30] Lacy BE , Patel NK. Rome criteria and a diagnostic approach to irritable bowel syndrome. J Clin Med. 2017;6:99. 29072609 10.3390/jcm6110099 PMC5704116

[31] O’Leary MP , Sant GR , Fowler FJ Jr , Whitmore KE , Spolarich-Kroll J. The interstitial cystitis symptom index and problem index. Urology. 1997;49 (5A Suppl):58–63. doi: 10.1016/s0090-4295(99)80333-1. 9146003

[32] DeRogatis L , Clayton A , Lewis-D’Agostino D , Wunderlich G , Fu Y. Validation of the female sexual distress scale–Revised for assessing distress in women with hypoactive sexual desire disorder. J Sex Med. 2008;5:357–364. 18042215 10.1111/j.1743-6109.2007.00672.x

[33] Jones G , Kennedy S , Barnard A , Wong J , Jenkinson C. Development of an endometriosis quality-of-life instrument: The Endometriosis health profile-30. Obstet Gynaecol. 2001;98:258–264. 10.1016/s0029-7844(01)01433-8 11506842

[34] Accuro Cloud [Internet]. QHR Technologies. 2024. (https://accuro.cloud) Accessed August 11, 2025.

[35] IBM Corp. IBM SPSS Statistics for Mac, Version 28.0. Armonk, NY: IBM Corp, 2021.

[36] Cohen J. A power primer. Psychol Bull. 1992;112:155–159. doi: 10.1037/0033-2909.112.1.155. 19565683

[37] Makhni EC , Higgins JD , Hamamoto JT , et al. Patient compliance with electronic patient-reported outcomes following shoulder arthroscopy. Arthroscopy. 2017;33:1940–1946. doi: 10.1016/j.arthro.2017.06.016. 28958797

[38] Patt D , Wilfong L , Hudson KE , et al. Implementation of electronic patient-reported outcomes for symptom monitoring in a large multisite community oncology practice: Dancing the Texas two-step through a pandemic. JCO Clin Cancer Inform. 2021;5:615–621. doi: 10.1200/CCI.21.00063. 34085537

[39] Sun EY , Alvarez C , Callahan LF , Sheikh SZ. The disparities in patient portal use among patients with rheumatic and musculoskeletal diseases: Retrospective cross-sectional study. J Med Internet Res. 2022;24:e38802. doi: 10.2196/38802. 36001872 PMC9439379

[40] Yu H , Yu Q , Nie Y , et al. Data quality of longitudinally collected patient-reported outcomes after thoracic surgery: Comparison of paper- and web-based assessments. J Med Internet Res. 2021;23:e28915. doi: 10.2196/28915. 34751657 PMC8663677

[41] Aanes SG , Wiig S , Nieder C , Haukland EC. Implementing digital patient-reported outcomes in routine cancer care: Barriers and facilitators. ESMO Real World Data Digit Oncol. 2024;6:100088. doi: 10.1016/j.esmorw.2024.100088. PMC12836584 41646091

[42] Wiegel J , Seppen BF , Nurmohamed MT , et al. Predictors for response to electronic patient-reported outcomes in routine care in patients with rheumatoid arthritis: A retrospective cohort study. Rheumatol Int. 2023;43:651–657. doi: 10.1007/s00296-023-05278-6. 36715728 PMC9885920

[43] Printza A. Patient-reported outcome measures in diseases of the head and neck. J Clin Med. 2022;11:3358. 35743429 10.3390/jcm11123358 PMC9224898

[44] Colls J , Lee YC , Xu C , et al. Patient adherence with a smartphone app for patient-reported outcomes in rheumatoid arthritis. Rheumatology. 2021;60:108–112. doi: 10.1093/rheumatology/keaa202. 32572490

[45] Jeong D , Aggarwal S , Robinson J , et al. Exhaustive or exhausting? Evidence on respondent fatigue in long surveys. J Dev Econ. 2023;161:102992. doi: 10.1016/j.jdeveco.2022.102992.

[46] Althubaiti A. Information bias in health research: Definition, pitfalls, and adjustment methods. J Multidiscip Healthc. 2016;9:211–217. doi: 10.2147/JMDH.S104807. 27217764 PMC4862344

[47] Kelley K , Clark B , Brown V , Sitzia J. Good practice in the conduct and reporting of survey research. Int J Qual Health Care. 2003;15:261–266. 12803354 10.1093/intqhc/mzg031

[48] Tang L , He Y , Pang Y , et al. Implementing symptom management follow-up using an electronic patient-reported outcome platform in outpatients with advanced cancer: Longitudinal single-center prospective study. JMIR Form Res. 2022;6:e21458. doi: 10.2196/21458. 35536608 PMC9131147

[49] Dupuis M , Strippoli MF , Gholam-Rezaee M , et al. Mental disorders, attrition at follow-up, and questionnaire non-completion in epidemiologic research: Illustrations from the CoLaus|PsyCoLaus study. Int J Methods Psychiatr Res. 2019;28:e1805. doi: 10.1002/mpr.1805. 31568629 PMC7027429

[50] Ghafourifard M. Survey fatigue in questionnaire-based research: The issues and solutions. J Caring Sci. 2024;13:214–215. doi: 10.34172/jcs.33287. 39974826 PMC11833437

[51] Rolstad S , Adler J , Rydén A. Response burden and questionnaire length: Is shorter better? A review and meta-analysis. Value Health. 2011;14:1101–1108. doi: 10.1016/j.jval.2011.06.003. 22152180

[52] Shepherdson P , Miller J. Redundancy gain in semantic categorization. Acta Psychol (Amst). 2014;148:96–106. doi: 10.1016/j.actpsy.2014.01.011. 24508611

