# Supplementary material for: Establishing an electronic patient-reported outcome (ePRO) for patients with endometriosis and chronic pelvic pain: A pilot feasibility study
Source: J Clin Transl Sci. 2025 Sep 29;9(1):e246. doi: 10.1017/cts.2025.10155 (PMC12695500; doi:10.1017/cts.2025.10155)
Supplement: Randle et al. supplementary material [file S2059866125101556sup001.docx]

**Supplementary Table 1.** Independent Samples t-Test Results for Non-Significant Demographic and Clinical Variables by Completion Experience (**Completed All Questions**)

| **Demographic/**  **Clinical Variable** | **Usability variable:**  **Completed all questions** | | **Mean Diff** | **95% CI**  **for mean diff** | | ***t (df)*** | ***p*** | **Effect Size** Hedge’s g | **95% CI**  **for g** | |
| --- | --- | --- | --- | --- | --- | --- | --- | --- | --- | --- |
|  | **Yes (n = 8)**  Mean ± | **No (n=3)**  Mean ± |  | Lower | Upper |  |  |  | Lower | Upper |
| Age | 34.38 ± 9.5 | 35.67 ± 10.9 | 1.29 | -20.69 | 23.28 | 0.18 (9) | .868 | 0.120 | -1.09 | 1.33 |
| PCS | 35.38 ± 13.1 | 21.0 ± 16.4 | -14.37 | -35.60 | 6.85 | -1.53 (9) | .160 | -0.94 | -2.21 | .36 |
| SF-MPQ Total* | 27.25 ± 8.0 | 24.0 ± 7.1 | -3.25 | -17.68 | 11.18 | -0.52 (8) | .618 | -0.37 | -1.76 | 1.05 |
| SF-MPQ Sensory* | 19.75 ± 4.7 | 15.5 ± 3.5 | -4.25 | -12.60 | 4.10 | -1.17 (8) | .274 | -0.83 | -2.26 | 0.64 |
| SF-MPQ Affective* | 7.5 ± 3.5 | 8.5 ± 3.5 | 1.00 | -5.33 | 7.33 | 0.36 (8) | .725 | 0.26 | -1.15 | 1.65 |
| TSK | 45.13 ± 7.0 | 38.33 ± 3.1 | -6.79 | -16.55 | 2.96 | -1.57 (9) | .150 | -0.97 | -2.24 | 0.34 |
| PSOCQ (pre-contemplation) | 3.66 ± 0.8 | 2.86 ± 0.5 | -0.80 | -1.94 | 0.34 | -1.58 (9) | .148 | -0.98 | -2.24 | 0.33 |
| PSOCQ (contemplation) | 3.87 ± 0.3 | 3.66 ± 0.4 | -0.20 | -0.72 | 0.31 | -0.90 (9) | .389 | -0.56 | -1.78 | 0.69 |
| PSOCQ (action) | 3.77 ± 0.3 | 3.88 ± 1.0 | 0.11 | -0.27 | 0.50 | 0.67 (9) | .517 | 0.41 | -0.82 | 1.63 |
| PSOCQ (maintenance) | 3.60 ± 0.7 | 3.95 ± 0.2 | 0.34 | -0.62 | 1.31 | 0.80 (9) | .442 | 0.49 | -0.74 | 1.71 |
| PHQ-9 | 18.38 ± 8.1 | 8.0 ± 5.2 | -10.37 | -21.97 | 1.22 | -2.02 (9) | .074 | -1.25 | -2.56 | -0.11 |
| ICSI | 8.63 ± 4.0 | 4.33 ± 3.2 | -4.29 | -10.16 | 1.58 | -1.65 (9) | .133 | -1.02 | -2.29 | 0.30 |
| ICPI | 6.88 ± 4.2 | 2.0 ± 3.5 | -4.87 | -11.10 | 1.35 | -1.77 (9) | .110 | -1.09 | -2.38 | 0.24 |
| FSDS-R ^#^ | 31.25 ± 14 | 17 ± 12.3 | -14.25 | -37.94 | 9.44 | -1.64 (9) | .172 | -0.95 | -2.21 | 0.35 |
| EHP-30 | 64.81 ± 18.8 | 45.9 ± 22.5 | -18.91 | -49.14 | 11.32 | -1.41 (9) | .191 | -0.87 | -2.13 |  |

Pain Catastrophizing Scale (PCS), Short-Form McGill Pain Questionnaire (SF-MPQ), Tampa Scale for Kinesiophobia (TSK), Pain Stages of Change Questionnaire (PSOCQ), Patient Health Questionnaire (PHQ-9), Interstitial Cystitis Symptom Index (ICSI), Interstitial Cystitis Problem Index (ICPI), Female Sexual Distress Scale–Revised (FSDS-R), Endometriosis Health Profile-30 (EHP-30).

*Group responding “No” n = 2 (one participant had missing data because the score could not be tabulated due to branching). **equal variances not assumed

**Supplementary Table 2.** Independent Samples t-Test Results for Non-Significant Demographic and Clinical Variables by Completion Experience (**number of sittings needed to complete the survey**).

| **Demographic/**  **Clinical Variable** | **Usability variable:**  **Number of sittings** | | **Mean Diff** | **95% CI**  **for mean diff** | | ***t (df)*** | ***p*** | **Effect Size** Hedge’s g | **95% CI**  **for g** | |
| --- | --- | --- | --- | --- | --- | --- | --- | --- | --- | --- |
|  | **1 sitting (n=7)** Mean ± | **>1 sitting (n=4)**  Mean ± |  | Lower | Upper |  |  |  | Lower | Upper |
| Age | 31.00 ± 8.92 | 41.25 ± 6.60 | -10.25 | -21.91 | 1.14 | -1.98 (9) | .780 | -1.39 | -2.35 | 0.12 |
| PCS | 32.71 ± 16.04 | 29.25 ± 14.19 | 3.46 | -18.45 | 25.38 | 0.37 (9) | .729 | 0.20 | -0.92 | 1.32 |
| SF-MPQ Total* | 14.19 ± 8.93 | 23.25 ± 3.86 | 5.58 | -5.50 | 16.66 | 1.16 (8) | .279 | 0.67 | -0.53 | 1.84 |
| SF-MPQ Sensory* | 20.17 ± 5.15 | 17.00 ± 3.65 | 3.16 | -3.75 | 10.08 | 1.05 (8) | .322 | 0.61 | -0.58 | 1.77 |
| SF-MPQ Affective*^#^ | 8.67 ± 4.08 | 6.25 ± 0.50 | 2.41 | -1.86 | 6.69 | 1.43 (8) | .209 | 0.67 | -0.53 | 1.84 |
| TSK | 43.29 ± 5.67 | 43.25 ± 9.53 | 0.03 | -10.16 | 10.24 | 0.01 (9) | .994 | 0.01 | -1.11 | 1.12 |
| CSI | 62.29 ± 20.93 | 57.75 ± 21.96 | 4.53 | -25.63 | 34.71 | 0.34 (9) | .742 | 0.19 | -0.93 | 1.31 |
| PSOCQ (precontemplation) | 3.59 ± 0.82 | 3.17 ± 0.80 | 0.41 | -0.73 | 1.57 | 0.81 (9) | .436 | 0.46 | -0.68 | 1.59 |
| PSOCQ (contemplation) | 3.81 ± 0.34 | 3.82 ± 0.37 | -0.01 | -0.51 | 0.49 | -0.04 (9) | .963 | -0.02 | -1.15 | 1.09 |
| PSOCQ (action) | 3.76 ± .28 | 3.87 ± .15 | -0.11 | -0.47 | 0.24 | -0.71 (9) | .490 | -0.41 | -1.53 | 0.73 |
| PSOCQ (maintenance) | 3.57 ± .74 | 3.92 ± .24 | -0.35 | -1.24 | 0.53 | -0.90 (9) | .391 | -0.51 | -1.65 | 0.64 |
| PHQ-9 | 18.14 ± 7.15 | 11.00 ± 10.23 | 7.14 | -4.63 | 18.91 | 1.37 (9) | .203 | 0.78 | -0.41 | 1.94 |
| GAD-7 | 12.86 ± 7.51 | 7.00 ± 5.09 | 5.85 | -3.79 | 15.50 | 1.37 (9) | .203 | 0.78 | -0.41 | 1.94 |
| ICSI | 7.29 ± 4.11 | 7.75 ± 4.85 | -0.46 | -6.66 | 5.73 | -0.16 (9) | .869 | -0.09 | -1.21 | 1.02 |
| ICPI | 5.00 ± 4.04 | 6.50 ± 5.68 | -1.50 | -8.10 | 5.10 | -.051 (9) | .620 | -0.29 | -1.41 | 0.84 |
| FSDS-R ^#^ | 30.86 ± 7.94 | 21.25 ± 22.39 | 9.60 | -24.76 | 43.98 | 0.82 (9) | .461 | 0.60 | -0.56 | 1.74 |
| EHP-30 | 63.12 ± 23.13 | 53.57 ± 16.79 | 9.55 | -20.54 | 39.65 | 0.71 (9) | .491 | 0.41 | -0.73 | 1.53 |

Pain Catastrophizing Scale (PCS), Short-Form McGill Pain Questionnaire (SF-MPQ), Tampa Scale for Kinesiophobia (TSK), Central Sensitization Inventory (CSI), Pain Stages of Change Questionnaire (PSOCQ), Patient Health Questionnaire 9 (PHQ-9), Generalized Anxiety Disorder 7 (GAD-7), Interstitial Cystitis Symptom Index (ICSI), Interstitial Cystitis Problem Index (ICPI), Female Sexual Distress Scale–Revised (FSDS-R), Endometriosis Health Profile-30 (EHP-30).

*Group responding “No” n = 2 (one participant had missing data because the score could not be tabulated due to branching). ^#^ Equal variances not assumed

**Supplementary Table 3.** Independent Samples t-Test Results for Non-Significant Demographic and Clinical Variables by Completion Experience (**time needed to complete** **the survey**)

| **Demographic/**  **Clinical Variable** | **Usability variable:**  **Time needed to complete** | | **Mean Diff** | **95% CI**  **for mean diff** | | ***t (df)*** | ***p*** | **Effect Size** Hedge’s g | **95% CI**  **for g** | |
| --- | --- | --- | --- | --- | --- | --- | --- | --- | --- | --- |
|  | ≤1 hour (n=6) Mean ± | >1 hour (n=4)  Mean ± |  | Lower | Upper |  |  |  | Lower | Upper |
| Age | 30.17 ± 9.47 | 41.25 ± 6.60 | -11.08 | -23.75 | 1.58 | -2.01 (8) | .078 | -1.17 | -2.42 | 0.12 |
| PCS | 29.83 ± 15.47 | 29.25 ± 14.19 | 0.58 | -21.75 | 22.92 | 0.06 (8) | .953 | 0.03 | -1.10 | 1.17 |
| SF-MPQ Total*^#^ | 27.00 ± 8.63 | 23.25 ± 3.86 | 3.75 | -6.91 | 14.41 | 0.86 (7) | .420 | 0.47 | -0.73 | 1.65 |
| SF-MPQ Sensory* | 19.00 ± 4.79 | 17.00 ± 3.65 | 2.00 | -4.88 | 8.88 | 0.68 (7) | .514 | 0.40 | -0.79 | 1.58 |
| SF-MPQ Affective*^#^ | 8.00 ± 4.18 | 6.25 ± .50 | 1.75 | -3.42 | 6.92 | 0.92 (7) | .405 | 0.48 | -0.72 | 1.66 |
| TSK | 43.67 ± 6.12 | 43.25 ± 9.53 | 0.41 | -10.87 | 11.70 | 0.08 (8) | .934 | 0.05 | -1.09 | 1.19 |
| CSI | 60.33 ± 22.22 | 57.75 ± 21.96 | 2.58 | -30.35 | 35.51 | 0.18 (8) | .861 | 0.10 | -1.04 | 1.24 |
| PSOCQ (precontemplation) | 3.64 ± .88 | 3.17 ± .80 | 0.46 | -0.80 | 1.74 | 0.84 (8) | .423 | 0.49 | -0.68 | 1.64 |
| PSOCQ (contemplation) | 3.71 ± .24 | 3.82 ± .37 | -0.10 | -0.55 | 0.34 | -0.55 (8) | .595 | 0.32 | -1.46 | 0.84 |
| PSOCQ (action) | 3.69 ± .24 | 3.87 ± .15 | -0.18 | -0.50 | 0.14 | -1.29 (8) | .231 | -0.75 | -1.93 | 0.46 |
| PSOCQ (maintenance) | 3.52 ± .80 | 3.92 ± .24 | -0.40 | -1.37 | 0.57 | -0.95 (8) | .370 | -0.55 | -1.71 | 0.63 |
| PHQ-9 | 17.17 ± 7.30 | 11.00 ± 10.23 | 6.16 | -6.51 | 18.85 | 1.12 (8) | .295 | 0.65 | -0.55 | 1.81 |
| GAD-7 | 12.17 ± 7.98 | 7.00 ± 5.09 | 5.16 | -5.31 | 15.65 | 1.13 (8) | .289 | 0.66 | -0.54 | 1.82 |
| ICSI | 7.83 ± 4.21 | 7.75 ± 4.85 | 0.08 | -6.56 | 6.73 | 0.02 (8) | .978 | 0.01 | -1.12 | 1.15 |
| ICPI | 5.17 ± 4.40 | 6.50 ± 5.68 | -1.33 | -8.66 | 5.99 | -0.42 (8) | .686 | -0.24 | -1.38 | 0.91 |
| FSDS-R ^#^ | 31.83 ± 8.23 | 21.25 ± 22.39 | 10.58 | -23.57 | 33.18 | 0.90 (8) | .423 | 0.62 | -0.57 | 1.79 |
| EHP-30 | 62.25 ± 25.20 | 53.57 ± 16.79 | 8.67 | -24.70 | 42.05 | 0.60 (8) | .566 | 0.35 | -0.81 | 1.49 |

Pain Catastrophizing Scale (PCS), Short-Form McGill Pain Questionnaire (SF-MPQ), Tampa Scale for Kinesiophobia (TSK), Pain Stages of Change Questionnaire (PSOCQ), Central Sensitization Inventory (CSI), Patient Health Questionnaire 9 (PHQ-9), Generalized Anxiety Disorder 7 (GAD-7), Interstitial Cystitis Symptom Index (ICSI), Interstitial Cystitis Problem Index (ICPI), Female Sexual Distress Scale–Revised (FSDS-R), Endometriosis Health Profile-30 (EHP-30).

*Group responding “No” n = 2 (one participant had missing data because the score could not be tabulated due to branching). ^#^ Equal variances not assumed

**Supplementary Table 4.** Independent Samples t-Test Results for Non-Significant Demographic and Clinical Variables by Redundancy and Comfort **(perceived redundance** **of survey questions**).

| **Demographic/**  **Clinical Variable** | **Usability variable:**  **Redundancy** | | **Mean Diff** | **95% CI**  **for mean diff** | | ***t (df)*** | ***p*** | **Effect Size** Hedge’s g | **95% CI**  **for g** | |
| --- | --- | --- | --- | --- | --- | --- | --- | --- | --- | --- |
|  | Yes (n=7) Mean ± | No (n=4)  Mean ± |  | Lower | Upper |  |  |  | Lower | Upper |
| Age | 36.86 ± 9.37 | 31.00 ± 9.35 | -5.87 | -19.13 | 7.41 | -0.99 (9) | .344 | -0.57 | -1.70 | 0.59 |
| SF-MPQ Total* | 23.17±7.16 | 31.75±5.31 | 8.58 | -1.14 | 18.31 | 2.03 (8) | .076 | 1.18 | -0.12 | 2.43 |
| SF-MPQ Sensory* | 17.0±4.42 | 21.75±3.86 | 4.75 | -1.53 | 11.03 | 1.74 (8) | .120 | 1.01 | -0.25 | 2.23 |
| SF-MPQ Affective* | 6.17±3.12 | 10.00±2.16 | 3.83 | -0.33 | 8.00 | 2.11 (8) | .067 | 1.23 | -0.08 | 2.49 |
| TSK | 41.43 ± 6.07 | 46.50 ± 7.72 | 5.07 | -4.39 | 14.53 | 1.21 (9) | .256 | 0.69 | -0.48 | 1.84 |
| CSI | 59.71 ± 21.96 | 62.25 ±20.13 | 2.53 | -27.77 | 32.84 | 0.18 (9) | .854 | 0.10 | -1.01 | 1.22 |
| PSOCQ (pre-contemplation) | 3.32 ± 0.97 | 3.64 ± .040 | 0.31 | -0.85 | 1.49 | 0.61 (9) | .555 | 0.35 | -0.79 | 1.47 |
| PSOCQ (contemplation) | 3.70±0.30 | 4.02±0.33 | 0.32 | -0.11 | 0.76 | 1.67 (9) | .129 | 0.95 | -0.27 | 2.13 |
| PSOCQ (action) | 3.71±0.24 | 3.95±0.16 | 0.24 | -0.07 | 0.56 | 1.72 (9) | .118 | 0.99 | -0.24 | 2.17 |
| PSOCQ (maintenance) ^#^ | 3.57 ± 0.75 | 3.92 ± 0.18 | 0.35 | -0.34 | 1.06 | 1.19 (9) | .271 | 0.52 | -0.63 | 1.65 |
| PHQ-9 | 12.86 ± 9.40 | 20.25 ± 5.18 | 7.39 | -4.29 | 19.08 | 1.43 (9) | .186 | 0.82 | -0.38 | 1.98 |
| GAD-7 | 9.43 ± 8.34 | 13.00 ± 4.24 | 3.57 | -6.69 | 13.83 | 0.78 (9) | .451 | 0.45 | -0.70 | 1.58 |
| ICSI ^#^ | 7.86 ± 5.08 | 6.75 ± 2.21 | -1.10 | -7.26 | 5.04 | -0.50 (9) | .630 | -0.23 | -1.35 | 0.90 |
| ICPI | 6.00 ± 5.06 | 4.75 ± 3.77 | -1.25 | -7.44 | 4.94 | -0.42 (9) | .680 | -0.24 | -1.36 | 0.89 |
| FSDS-R | 24.00 ± 15.72 | 33.25 ± 11.78 | 9.25 | -11.35 | 29.85 | 1.01 (9) | .336 | 0.58 | -0.58 | 1.72 |

Short-Form McGill Pain Questionnaire (SF-MPQ), Tampa Scale for Kinesiophobia (TSK), Central Sensitization Inventory (CSI), Pain Stages of Change Questionnaire (PSOCQ), Patient Health Questionnaire 9 (PHQ-9), Generalized Anxiety 7 (GAD-7), Interstitial Cystitis Symptom Index (ICSI), Intersticial Cysticis Problem Index (ICPI), Female Sexual Distress Scale–Revised (FSDS-R).

*Group responding “No” n = 2 (one participant had missing data because the score could not be tabulated due to branching). ^#^ Equal variances not assumed
